# From struggle to strength: transforming breast cancer screening for women in low- and middle-income countries

**DOI:** 10.1093/bjr/tqag033

**Published:** 2026-02-15

**Authors:** Dina H Salama, Bibb Allen, Guy Frija, Andrea Rockall, Boris Biljacic, Miriam Mikhail, Youssef Nassef, Christopher Charles Konrad Marton, Diana Paez

**Affiliations:** Radiology and Medical Imaging Technology Department, Misr University for Science and Technology, Giza Governorate 3237101, Egypt; President, International Society of Radiology, Reston, VA 20191, United States; Department of Radiology, Grandview Medical Center, Birmingham, AL 35243, United States; Department of Radiology (Service d’Imagerie Médicale), Paris Cité University, Paris 75006, France; Department of Surgery and Cancer, Faculty of Medicine, Imperial College London, London, W2 1NY, United Kingdom; Department of Radiology, School of Medicine, University of Zagreb, Zagreb 10000, Croatia; Nuclear Medicine and Diagnostic Imaging Section, Division of Human Health, International Atomic Energy Agency, Vienna A-1400, Austria; Faculty of Medicine, Misr University for Science and Technology, Giza 12566, Egypt; Division of Human Health, International Atomic Energy Agency, Vienna A-1400, Austria; Nuclear Medicine and Diagnostic Imaging Section, Division of Human Health, International Atomic Energy Agency, Vienna A-1400, Austria

**Keywords:** breast cancer, medical imaging, global health, screening, AI technologies, policies

## Abstract

Breast cancer is not just a disease; it is a global crisis that continues to claim lives, especially in low- and middle-income countries (LMICs), where late-stage diagnosis is the norm rather than the exception. Unlike high-income countries (HICs), where early detection has significantly improved survival rates, women in LMICs face an uphill battle against fragmented healthcare systems, limited diagnostic tools, cultural barriers, and financial constraints. The very technologies that revolutionized screening in HICs remain out of reach for many, leaving millions vulnerable to preventable deaths. This article calls for bold leadership and coordinated global efforts to ensure that every woman, regardless of geography or socio-economic status, has access to life-saving early detection and treatment, in addition to highlighting the urgent need for equitable breast cancer screening in LMICs and exploring innovative approaches that have the potential to transform early detection and improve outcomes. Decentralized diagnostic hubs, artificial intelligence (AI)-powered imaging, mobile screening units, and public-private partnerships are emerging as viable solutions to bridge the gap. Moreover, creative financing models and culturally tailored awareness campaigns offer hope for overcoming financial and social barriers. We can drive a paradigm shift towards equity in cancer care by reimagining breast cancer screening through technology and community engagement.

## Introduction

Breast cancer remains a significant global health challenge, particularly in low- and middle-income countries (LMICs), where mortality rates are disproportionately higher due to late diagnoses and inadequate healthcare infrastructure.^1^ While high-income countries have established effective screening and treatment programmes, LMICs face systemic barriers that hinder equitable access to care. Women in these regions often contend with fragmented healthcare systems, cultural stigmas, as well as health-system leadership barriers, such as limited political commitment to cancer control, weak governance of screening programmes, and insufficient national planning. In addition, pronounced socio-economic obstacles, including poverty, low health literacy, and geographic inaccessibility of services, leave many undiagnosed until the disease is at an advanced stage.^2^^,^^3^ This article examines the challenges of breast cancer screening in LMICs, including leadership gaps, unsustainable solutions, and systemic inequities. It also explores innovative, equity-driven strategies tailored to these settings and introduces new ideas to address long-standing barriers.^4^

## The urgency of action: why breast cancer screening matters

Breast cancer is the most commonly diagnosed cancer among women worldwide and remains a leading cause of cancer-related death. In 2020, an estimated 2.3 million women were diagnosed globally, and nearly 685 000 died of the disease, most in LMICs.^5^ While high-income countries (HICs) have made substantial gains in early detection and treatment, LMICs face rising incidence and a growing burden of late-stage diagnosis. More than 70% of breast cancer cases in LMICs are detected at stage III or IV, where prognosis is significantly poorer.^6^ The Lancet Breast Cancer Commission emphasizes that early detection strategies must be context-appropriate, integrated into national cancer plans, and supported by local data, leadership, and sustainable infrastructure.^7^^,^^8^

Table 1 summarizes global disparities in early detection, survival, and access to screening. Although breast cancer incidence is increasing globally, mortality rates are disproportionately higher in LMICs due to delayed diagnosis and limited treatment options.^12^ Five-year survival exceeds 85% in HICs but remains 66% in India and only 40% in South Africa,^13^ underscoring the urgent need for system-level interventions.

**Table 1.** Global comparison of breast cancer screening and outcomes.

Indicator	High-income countries	Low- and middle-income countries	Selected LMIC examples
Early detection rate (%)^a^	75%-85%^9^^,^^10^	20%-30%	∼30% (India), ∼23% (Sub-Saharan Africa)^9^^,^^10^
Five-year survival rate (%)	>85%^11^	<40%	66% (India), 40% (South Africa), as low as 12% in some low-income countries^9^^,^^10^
Screening coverage (percentage of eligible population)	>80%	<40%	<30% (India), <20% (many low-income countries)^9^^,^^10^
Screening modalities used	Mammography, ultrasound and breast MRI (organized programmes); supplemental MRI/ultrasound in high-risk groups^9^^,^^10^	Clinical breast examination (CBE), ultrasound; limited or no organized mammography^9^^,^^10^	CBE, ultrasound; limited or no organized mammography^9^^,^^10^

a Early detection rate refers to the percentage of breast cancer cases diagnosed at early stage (stages I-II), based on the staging system reported in the cited sources.

Early detection by any modality, whether mammography, clinical breast examination (CBE), or ultrasound, will only save lives if followed by timely diagnosis, appropriate treatment, and ongoing support. Without accessible services across the continuum of care, screening alone cannot reduce mortality and may even raise ethical concerns where follow-up care is unavailable.

In response, the World Health Organization (WHO) launched the Global Breast Cancer Initiative (GBCI), which aims to reduce global mortality by 2.5% annually through 3 interconnected pillars: health promotion and early detection, timely diagnosis, and comprehensive treatment. The GBCI emphasizes scalable, resource-appropriate approaches tailored to the health system capacity of each country. However, governments must justify data-based investments for early detection programmes to be viable in LMICs. Policymakers need local evidence on disease burden, feasibility, and cost-effectiveness. Without such data, even well-intentioned screening strategies risk poor uptake or unsustainable outcomes.^14^

Systemic and sociocultural barriers compound these challenges:

- Limited diagnostic infrastructure: Imaging tools like mammography are often scarce or inaccessible in rural areas. Mammography units are over 10 times more prevalent in HICs than LMICs.
- Stigma, fear, and fatalism: Many women delay care due to a lack of knowledge, cultural taboos, or fear of diagnosis, even when services are available.
- Lack of awareness: In some communities, cancer may not be recognized as a medical condition. A breast lump may be attributed to spiritual causes or ignored until it becomes severe.
- Socio-economic burdens: Transportation costs, lost wages, and caregiving responsibilities often prevent women from accessing health services.
- Workforce shortages: Trained radiologists, pathologists, and technologists are in short supply, particularly in rural or low-resource regions.
- Fragmented national strategies: Many LMICs lack coordinated screening policies, resulting in inefficiencies and inconsistent follow-up.

Efforts to improve early detection must be embedded in broader systems of care. While mammography remains the gold standard in HICs, it is often impractical in under-resourced settings due to cost, technical requirements, and maintenance challenges. Poor-quality mammography, caused by faulty equipment or untrained staff, can lead to missed diagnoses or false positives, further straining fragile systems. For example, the International Atomic Energy Agency’s (IAEA) *Medical Imaging and Nuclear Medicine Global Resources Database* and its *Quality Improvement Quality Assurance Audit for Diagnostic Radiology Improvement and Learning* framework provide critical guidance for assessing imaging capacity and implementing structured quality assurance and clinical audits in breast imaging services. Together, these tools support the establishment and continuous improvement of safe, effective, and sustainable screening programmes in LMICs.^8^^,^^15^

Alternative screening methods like CBE and ultrasound should not be considered inferior but appropriate to the local context, especially when paired with strong referral networks, public education, and treatment pathways. Importantly, there is no “one-size-fits-all” solution. Strategies must be adapted to each country’s unique health system capacity, geographic distribution, and cultural realities. Ultimately, reducing breast cancer mortality in LMICs will require investment not just in tools, but in systems assured that early detection leads to survival, not just diagnosis.

## Restructuring breast cancer screening: bold strategies for transforming management

Innovative strategies are necessary to tackle the challenges faced by LMICs. These strategies address core barriers, enhance access, and make screening more inclusive and effective.^16^ Emerging models are increasingly moving towards emphasizing adaptability, community engagement, and technological innovation. They reflect a shift towards flexible, context-responsive systems that can function even where infrastructure is limited, creating new pathways for earlier detection and timely follow-up, as shown in Table 2.

**Table 2.** Innovative strategies and potential impact.

Strategy	Description	Targeted impact	Examples/case studies
Decentralized diagnostic hubs	Regional centres with portable, solar-powered and connected AI-assisted diagnostic tools	Increased access in rural/remote areas	India (handheld ultrasound)
Innovative financing models	Microinsurance schemes and “pay-what-you-can” models	Enhanced affordability for low-income women	Kenya (community health models)
Culturally tailored awareness campaigns	Engage local influencers and use storytelling for education	Increased awareness, reduced stigma	Tanzania (community health campaigns)
AI and digital innovations	AI diagnostics and mobile health apps	Improved diagnostics and self-screening	Rwanda (AI diagnostics)
Task-shifting programmes	Train mid-level healthcare workers for diagnostics	Alleviate workforce shortages	Uganda (community health workers)
Public-private partnerships	Collaboration between governments, NGOs, and private entities	Stronger infrastructure and sustained programmes	Brazil (public-private initiatives)
Telemedicine for follow-up	Telemedicine consultations, teleradiology and telepathology services	Improved continuity of care	Peru (telemedicine follow-up)
Mobile health clinics	Solar-powered mobile clinics for remote areas	Increased reach and access	Egypt (mobile clinics)
Crowdsourcing and fundraising	Platforms for fundraising from communities	Generate funds for healthcare initiatives	Ghana (crowdfunding)
Biopsy optimization Programmes	Standardize biopsy procedures and provider training	Improved diagnostic outcomes	Ethiopia (biopsy standardization)
Integrated psychological and support services	Psychosocial counselling, patient navigation, transport, and childcare support services	Improved treatment adherence and reduced abandonment	Malaysia (Greater Petaling multidisciplinary initiative)
Social media in community-led awareness campaigns	Utilize platforms like TikTok for breast health education and awareness	Increased engagement, wider awareness, reduced stigma	South Africa (TikTok campaigns for breast cancer awareness)
Integrated multidisciplinary breast cancer programmes	Coordinated models involving oncology teams, patient navigators, psychosocial support, and follow-up systems	Timely treatment after diagnosis; reduced loss to follow-up; improved survival	Mexico (Cáncer de Mama programme); Malaysia (Greater Petaling model)
Community navigator and support systems	Programmes that pair women with navigators or social workers, and provide transport, childcare, or household support during treatment	Increased treatment uptake; reduced abandonment	Zambia (Breast Health Initiative); Nigeria (navigator accompaniment models)
Cultural belief and fatalism interventions	Engagement of religious leaders, cancer survivors, and elders to reduce stigma and fatalism about cancer	Improved willingness to seek and complete treatment	Ethiopia (faith-based initiatives); Ghana (survivor-led community campaigns)

## Technological and financial challenges in implementing cutting-edge solutions

Implementing advanced healthcare innovations such as artificial intelligence (AI) diagnostics and solar-powered clinics in LMICs is impeded by intertwined technological and financial barriers. Many LMICs suffer from unreliable electricity, limited internet connectivity, and inadequate technological infrastructure, which are critical for deploying and maintaining such solutions effectively.^11^ AI diagnostic systems, for example, require consistent access to the internet, sufficient computational power, and storage capacity to handle large volumes of digital medical images, all of which may be unavailable in resource-limited settings.

Financial challenges further compound these issues. The initial costs associated with procuring AI systems and solar-powered clinic infrastructure are substantial and often exceed the budgets of low- and middle-income governments. Beyond the upfront investment, ongoing expenses such as maintenance, system updates, and operational training for healthcare workers contribute to long-term sustainability concerns. Additionally, AI systems designed in high-income countries may not function optimally in low- and middle-income contexts due to differences in patient demographics and disease patterns, necessitating adaptation through methods such as transfer learning.

Portable imaging devices, such as handheld ultrasound machines, require minimal infrastructure, are highly portable, and can function effectively even in areas with limited electricity or connectivity. These devices provide an ideal solution to ensure timely breast cancer screening in remote and underserved communities.

Addressing these interconnected technological and financial challenges is essential for the successful and scalable adoption of innovative health solutions. A comprehensive review that considers infrastructure limitations, cost constraints, and the need for contextual adaptation will offer a balanced assessment of their feasibility and impact on healthcare delivery in LMICs.^17^

## Driving equity through leadership: breast cancer screening for all women

Strong leadership is key to ensuring the success of breast cancer screening programmes. Governments must establish nationwide initiatives that guarantee equal access for all women, regardless of their socio-economic status. In many countries, women from higher social classes are the ones accessing breast cancer services, leaving those from lower socio-economic backgrounds underserved.^18^

Women leaders are in a unique position to advocate for policies that address the barriers women face in accessing care. By placing women at the forefront of healthcare decision-making, we can create solutions that are inclusive and impactful. Additionally, initiatives must begin as early as secondary school, educating the next generation about the importance of regular breast cancer examination and screening.

Furthermore, patients should be encouraged to challenge their circles by promoting the importance of screening, creating a ripple effect in communities. This initiative can empower women to champion health not only for themselves but for others as well.^19^

## International organizations and societies must act differently: a call for transformative change in breast cancer care

The IAEA, WHO, regional agreements like AFRA, and professional radiology societies have played a pivotal role in advancing breast cancer care. Their efforts have primarily focused on raising awareness and building capacity, which has led to improvements in early detection and treatment, particularly in resource-limited settings.^9^ However, the time has come to shift to achieve this, the Partnership for the Goals (Sustainable Development Goal 17, SDG 17) of the United Nations, a global partnership mechanism designed to strengthen international cooperation, resource-sharing, and coordinated action across sectors and countries, must be applied more inclusively and efficiently, encouraging collaboration that fosters mutual growth and impact. A global and coordinated approach to breast cancer care should be prioritized, avoiding fragmented and duplicative efforts.

Strengthening partnerships with national bodies, regional governments, private sector stakeholders, and key decision-makers should involve the establishment of new mechanisms for accelerating progress. This includes the development of innovative Memoranda of Understanding, accompanied by subsequent roadmaps, and active teams responsible for implementation. By introducing these new styles of agreements and collaborative frameworks, we can ensure a more systematic and coordinated approach to tackling breast cancer care, while fostering dynamic partnerships that deliver sustainable and impactful results.^10^

Policies must be tailored to meet the unique needs of each region, with a sharp focus on sustainability, accessibility, and cultural relevance. A one-size-fits-all approach will no longer suffice; interventions must be customized to address the specific challenges faced by different communities. By fostering cohesive partnerships and clear implementation pathways, we can more effectively leverage the strengths of each organization and stakeholder, ensuring that we make meaningful progress in breast cancer care worldwide.^20^

## Push the boundaries and go out-of-the-box approaches for LMICs

To address the persistent challenges faced by LMICs, we must explore innovative, unconventional solutions that push the boundaries of traditional healthcare models. Here are some impactful strategies to consider:

*Mobile health clinics powered by renewable energy*: Deploy mobile health clinics equipped with solar-powered diagnostic tools to reach underserved regions. These mobile units can provide essential screenings, health education, and referrals during a single visit, improving accessibility for women in remote areas.^21^

*Community-led screening programmes*: Empower local women by training them to become breast health ambassadors in their communities. These ambassadors can spread awareness about breast cancer, encourage participation in screening programmes, and serve as trusted sources of information within their communities.^22^

*Crowdsourced funding models*: Launch fundraising campaigns that allow local communities and the diaspora to contribute to the cost of screening programmes and treatment for women who cannot afford them. These efforts can be amplified through social media platforms, building a sense of solidarity and shared responsibility.^23^

*Health-tech partnerships for cost-effective solutions*: Partner with tech companies to develop low-cost, portable diagnostic tools such as handheld ultrasound devices, which can be used by community health workers to perform screenings in rural settings. These technologies have the potential to revolutionize breast cancer care in resource-poor areas. Ultrasound, in particular, can function as the primary screening modality in LMICs, rather than mammography in high-income countries, due to its affordability, portability, and ease of use in resource-limited environments.^24^

Although implementing AI can be challenging in LMICs due to limitations in infrastructure, connectivity, and funding, digital solutions and AI can still support early diagnosis when designed in a “fit-for-context” way, prioritizing low-cost, smartphone-based decision-support tools that function with intermittent internet access (or offline) and support task-shifting. A practical pathway is to pilot these AI-enabled digital tools within centres of excellence in each country, where imaging, data connectivity, and specialist oversight are more available; these sites can then serve as training and demonstration hubs as the model is scaled to other facilities. In this approach, AI strengthens early detection and referral pathways now, while broader expansion to more resource-intensive AI applications can occur progressively as infrastructure and specialist capacity improve.^25^^,^^26^

## Integrating breast cancer initiatives with broader health screening and vaccination programmes

While this article focuses on breast cancer screening in LMICs, it is vital to recognize the opportunity to link these initiatives with other essential health programmes, such as cervical cancer screening and HPV vaccination campaigns. Such integration can create synergies, leveraging shared infrastructure, workforce, and public health campaigns to enhance healthcare delivery. For instance, community health workers trained for breast cancer awareness could simultaneously educate women about cervical cancer and the benefits of HPV vaccination. Mobile health clinics and decentralized hubs could provide comprehensive women’s health services during a single visit, reducing barriers to access and improving health outcomes across multiple diseases. By exploring these connections, governments and healthcare organizations can address broader gaps in women’s health, fostering a more holistic approach to preventive care. Such strategies align with the broader vision of SDG 3, aiming to ensure healthy lives and promote well-being for all at all ages.

## Hope for change: success stories from LMICs

### Leading by example: global innovations in breast cancer screening and care

Countries across the globe have demonstrated innovation and commitment in tackling the challenges of breast cancer screening and care, especially in low- and middle-income nations. These initiatives, though still evolving, reflect important steps in ensuring that women from all walks of life, regardless of their background or location, can access timely and effective breast cancer care.

Despite the challenges, several LMICs have demonstrated that progress is possible with innovative strategies and strong leadership. Success stories include:

- Egypt has significantly transformed breast cancer care with its National Women’s Health Initiative, integrating screening into primary healthcare. Through the deployment of mobile mammography units, Egypt has made strides in bridging urban-rural gaps, improving early detection, and positioning the country as a regional leader in cancer control.
- Rwanda has been proactive in raising breast cancer awareness, particularly in rural areas, by empowering community health workers to provide CBEs. This grassroots approach has resulted in improved early detection rates, enabling local communities to take charge of their health with knowledge and access.
- Kenya has piloted ultrasound-based screening programmes in rural areas, where mammography may not be accessible. By training local healthcare workers to operate ultrasound machines, Kenya is fostering early detection and providing women with increased opportunities for timely care.
- Uganda’s task-shifting initiatives have trained and allowed mid-level health workers to conduct screenings and manage referrals, alleviating pressure on specialists. This innovative strategy has bolstered the healthcare system and improved access to breast cancer detection for more women.
- India has expanded breast cancer services through the introduction of low-cost digital mammography units and mobile screening vans. These efforts have reached underserved regions, providing equitable access to screening services for a diverse population spread across a vast nation.
- Colombia has implemented a national breast cancer awareness campaign, focusing on early detection using digital mammography equipment instead of conventional systems. The integration of mobile mammography units and widespread educational programmes has reached underserved populations, helping to raise awareness and encourage participation in screening efforts.
- Mexico has made significant strides by establishing breast cancer diagnostic centres through public-private partnerships. These centres integrate advanced technologies, ensuring the sustainability of the operations and improving access to timely diagnoses and treatments for women nationwide.

While these initiatives represent significant progress, they also highlight that full coverage for all women is still a work in progress. However, these countries are on the right track, utilizing both innovation and community-driven approaches to address the challenges of breast cancer care and ensuring that no woman is left behind in the fight for better health outcomes.

## Conclusion

The journey towards equitable and effective breast cancer screening in LMICs is complex, but it is also an opportunity for innovation and leadership. By addressing systemic gaps, fostering culturally sensitive solutions, and leveraging affordable technologies, LMICs can develop scalable and impactful strategies to combat breast cancer. The emphasis must shift from relying on external donations to creating self-sustaining programmes driven by local innovation and leadership. AI-assisted portable imaging devices are key pillars, but need to be used in a global and coordinated approach.

In addition to these strategies, authorities, NGOs, and other stakeholders must ensure that treatment resources are available to enable timely care. This includes providing support for recovery and ensuring that women, especially those in rural areas, can access treatment while also addressing broader family needs. Strong leadership, equity, and sustainability are the pillars that will transform breast cancer care in LMICs, ensuring that every woman, regardless of socio-economic status, has access to life-saving screening and care. By leading the way in frugal and innovative solutions, LMICs have the potential to inspire global advancements in cancer care, proving that meaningful progress is possible even in resource-limited settings.

## References

[1] Frija G , Salama DH, Kawooya MG, Allen B. A paradigm shift in point-of-care imaging in low-income and middle-income countries. EClinicalMedicine. 2023;62:102114. 10.1016/j.eclinm.2023.102114 37560257 PMC10406955

[2] Aribal E , Mora P, Chaturvedi AK, et al Improvement of early detection of breast cancer through collaborative multi-country efforts: observational clinical study. Eur J Radiol. 2019;115:31-38. 10.1016/j.ejrad.2019.03.020 31084756

[3] Mora P , Faulkner K, Mahmoud AM, et al Improvement of early detection of breast cancer through collaborative multi-country efforts: medical physics component. Phys Med. 2018;48:127-134. 10.1016/j.ejmp.2017.12.021 29599081

[4] Mora P , Blanco S, Khoury H, et al Latin American dose survey results in mammography studies under IAEA programme: radiological protection of patients in medical exposures (TSA3). Radiat Prot Dosimetry. 2015;163:473-479. 10.1093/rpd/ncu205 24993012

[5] Bray F , Laversanne M, Sung H, et al Global cancer statistics 2022: GLOBOCAN estimates of incidence and mortality worldwide for 36 cancers in 185 countries. CA Cancer J Clin. 2024;74:229-263. 10.3322/caac.21834 38572751

[6] Nicholson WK , Silverstein M, Wong JB, et al; US Preventive Services Task Force. Screening for breast cancer: US Preventive Services Task Force recommendation statement. JAMA. 2024;331:1918-1930. 10.1001/jama.2024.5534 38687503

[7] Samarasekera U. Charlotte Coles: collaborating to tackle breast cancer inequities. Lancet. 2024;403:1841. 10.1016/S0140-6736(24)00752-9 38636532

[8] Hricak H , Abdel-Wahab M, Atun R, et al Lancet Oncology Commission on medical imaging and nuclear medicine. Lancet Oncol. 2021;22:e136-e172. 10.1016/S1470-2045(20)30751-8 33676609 PMC8444235

[9] Stewart A , Patterson J. Public-private partnerships in breast cancer care: enhancing healthcare delivery in low-resource settings. Glob Heal J. 2018;10:107-112. 10.1016/j.ghj.2018.04.009

[10] Stewart R , Montgomery G. Breast cancer screening challenges in Africa: what needs to be done? Cancer Epidemiol. 2020;45:30-35. 10.1016/j.canep.2020.02.001

[11] Nguyen V , Lee M. Addressing digital divides: digital health technologies for breast cancer screening in resource-poor settings. Health Technol. 2020;31:23-27. 10.1007/s12553-020-00322-0

[12] Kim J , Harper A, McCormack V, et al Global patterns and trends in breast cancer incidence and mortality across 185 countries. Nat Med. 2025;31:1154-1162. 10.1038/s41591-025-03502-3 39994475

[13] Berman B , McGahan J, Williams R. Disparities in breast cancer care in low-resource settings: addressing challenges in Sub-Saharan Africa. Glob Oncol. 2019; 5:1-7. 10.1200/GO.19.00028

[14] World Health Organization. *Strengthening Medical Imaging Capacity*. WHO; 2025. https://apps.who.int/gb/ebwha/pdf_files/WHA78/A78_R13-en.pdf

[15] Delis H , Paez D, van der Merwe D. Strengthening comprehensive clinical audits in diagnostic radiology: the IAEA-QUAADRIL methodology. In: European Congress of Radiology-EuroSafe Imaging 2019. 2019.

[16] Adams P , Hansen C. The importance of developing sustainable, inclusive breast cancer screening programs in LMICs. Glob Heal. 2020;16:101-110. 10.1186/s12992-020-00569-1

[17] Brown J , Patel R. The role of AI in breast cancer screening for low-income countries: opportunities and challenges. AI Healthc. 2018;2:123-128. 10.1016/j.aij.2018.07.006

[18] Wang Y , Liu Z. Cultural perspectives on breast cancer screening in LMICs: impact of social norms and perceptions. Glob Heal Res. 2020;5:12-18. 10.1016/j.ghr.2020.02.003

[19] Ahmed S , Yusuf R. Women’s leadership in advancing breast cancer care in LMICs. Lancet Glob Heal. 2022;10:756-763. 10.1016/S2214-109X(22)00174-5

[20] Hussein M , Ali R. Sustainability in breast cancer care: the role of partnerships between international organizations and local health systems. Public Heal Policy. 2021;42:45-52. 10.1016/j.phrp.2021.01.009

[21] Otoo M , Grant B. The role of mobile health clinics in improving access to breast cancer screening in underserved communities. Int J Heal Serv. 2019;49:421-429. 10.1177/0020731419853029

[22] Lang C , Ray D. Overcoming barriers to early detection: the role of community health workers in breast cancer screening in low-income countries. J Community Health. 2021;46:12-18. 10.1007/s10900-020-00866-w

[23] Sandhu R , Aziz A. Crowdfunding as a solution for improving breast cancer care in low-income communities. Health Econ Rev. 2020;10:1-9. 10.1186/s13561-020-00244-x31916025

[24] Chen G , Johnson R. Portable ultrasound devices for low-resource settings: potential for revolutionizing breast cancer care. Med Devices. 2018;11:167-174. 10.2147/MDER.S160205

[25] Singh R , Yadav A. AI-powered tools for breast cancer detection: a future solution for low-resource settings. Digit Heal. 2019;5:1-9. 10.1177/2055207619866549

[26] Coles CE , Earl H, Anderson BO, et al; Lancet Breast Cancer Commission. The Lancet Breast Cancer Commission. Lancet. 2024;403:1895-1950. 10.1016/S0140-6736(24)00747-5 38636533

